# A comparison of missing data methods for hypothesis tests of the treatment effect in substance abuse clinical trials: a Monte-Carlo simulation study

**DOI:** 10.1186/1747-597X-3-13

**Published:** 2008-06-03

**Authors:** Sarra L Hedden, Robert F Woolson, Robert J Malcolm

**Affiliations:** 1 Department of Biostatistics, Bioinformatics and Epidemiology, Medical University of South Carolina, 135 Cannon Place, Charleston, SC 29425, USA; 2 Department of Psychiatry and Behavioral Research, Medical University of South Carolina, 67 President Street, Charleston, SC 29425, USA; 3 Department of Mental Health, Johns Hopkins Bloomberg School of Public Health, 2213 McElderry St., Suite 400, Baltimore, MD 21205, USA

## Abstract

**Background:**

Missing data due to attrition are rampant in substance abuse clinical trials. However, missing data are often ignored in the presentation of substance abuse clinical trials. This paper demonstrates missing data methods which may be used for hypothesis testing.

**Methods:**

Methods involving stratifying and weighting individuals based on missing data pattern are shown to produce tests that are robust to missing data mechanisms in terms of Type I error and power. In this article, we describe several methods of combining data that may be used for testing hypotheses of the treatment effect. Furthermore, illustrations of each test's Type I error and power under different missing data percentages and mechanisms are quantified using a Monte-Carlo simulation study.

**Results:**

Type I error rates were similar for each method, while powers depended on missing data assumptions. Specifically, power was greatest for the weighted, compared to un-weighted methods, especially for greater missing data percentages.

**Conclusion:**

Results of this study as well as extant literature demonstrate the need for standards of design and analysis specific to substance abuse clinical trials. Given the known substantial attrition rates and concern for the missing data mechanism in substance abuse clinical trials, investigators need to incorporate missing data methods a priori. That is, missing data methods should be specified at the outset of the study and not after the data have been collected.

## Background

Treatment delivery for substance abuse has evolved from inpatient care to intensive outpatient care [1]. Although outpatient settings have increased the population of participants able to receive treatment, attrition is substantial in outpatient substance abuse treatment settings. Recent studies of substance abuse treatment clinical trials demonstrate considerable drop-out after first dose of treatment [2-6].

The high percentage of study participant attrition documented in substance abuse research interferes with the effectiveness of treatment programs and calls into question the validity of study analyses. Furthermore, poor outcomes are associated poor treatment retention [7]. Although missing data are rampant, it is often ignored in the presentation of clinical trials [4,8] and statistical methods of longitudinal data analysis often used in the substance abuse literature, such as data deletion or single imputation, may be biased or otherwise invalidated in the presence of substantial missing data and/or when missing data that is not missing completely at random [8]. This is particularly true in substance abuse clinical trials where missing data in outcomes at a particular point in time may be dependent upon previous outcomes. For example, a participant is likely to drop out of a substance abuse treatment clinical trial at the time of relapse.

The statistical literature details many methods of longitudinal data analysis that handle missing data; many have demonstrated robustness to assumptions of the missing data mechanism. These methods include, but are not limited to, multiple imputation [9,10], pattern mixture models [11], selection models and stratified summary statistics (SSS) [12-16]. This article describes one of the methods, SSS, which may be used specifically for hypothesis testing of the treatment effect.

We first provide an overview of summary statistic and SSS methods. Next, we discuss modification and expansion of the SSS method using some of the methods often used in the statistical literature for data combination. Comparisons of these methods are made under different assumptions for the missing data – both mechanism and rate of attrition. Finally, we conclude by describing some of the strengths and limitations of SSS methods.

### Summary Statistic Methods of Longitudinal Data Analysis and Missing Data

The summary statistic method of longitudinal data analysis is a technique by which each participant's multivariate outcome is reduced to a scalar summary measure. Comparisons of the scalar summary measures between treatments may then be analyzed using a variety of univariate statistical techniques [12,15,17-19]. For example, a summary statistic (e.g. mean, slope) is calculated for each individual over time. Then the average summary statistic response for each treatment group is calculated and compared using an independent t-test.

As with any type of longitudinal data analysis, the summary statistic approach may need to be modified for losses to follow-up. Dawson and Han [14] studied the effect of missing data mechanism on summary statistics. For example, when the slope is used as a summary statistic and the missing data mechanism is considered to be completely random (MCAR) the *variance *of the slopes varies dependent on the amount of outcome data available [14]. However, if the missing data mechanism is missing at random or nonignorable (MNAR) and/or the trend is nonlinear then the *mean *of the slopes may vary dependent upon the amount of information available per individual.

If the missing data patterns differ between treatment arms, the summary test statistic approach may be invalid [20]. A method proposed by Dawson [12-16] may be applied to a variety of summary statistics whereby each participant's summary response is stratified according to their missing data pattern. This method is called Stratified Summary Statistic (SSS) as one stratifies the analysis according to missingness patterns. This 'stratification by missingness pattern' may be appropriate when the mean and/or variance of the summary statistic is dependent upon the amount or timing of the outcome [12-16].

The computation of SSS as described by Dawson [13] is detailed below.

#### Stratified Summary Statistic Calculations

(1) Define an appropriate scalar measure (summary statistic) of the multivariate outcome (e.g. slope, mean,) and compute the summary statistic for each individual over time. For example, when an outcome is expected to linearly increase or decrease over time, a slope may be a good selection of a summary statistic [13]. Statistically, the slopes, *S*_*sj *_may be calculated for each participant over time, s = 1,..., *t *in each treatment group, j = 1, 2.

(2) Stratify participant slopes by the missing data pattern; slopes are stratified by the timing of each participant's dropout, *s *= 1,..., *t*. For example, slopes in which subjects had two observations over time will be placed in one stratum; whereas, slopes in which subjects had three observations over time will be placed in a separate stratum, etc

(3) Compute stratum-specific test statistics, e.g., a t-test comparing average treatment differences. Suppose that the null hypothesis of interest is to test whether the distribution functions of *S*_*s*1 _and *S*_*s*2 _are equal, *H*_*o*_: *F*_*s*1 _(*s*) = *F*_*s*2 _(*s*). Once slopes are calculated for each individual in each treatment arm, a stratum specific t-test may be defined where independent observations are available and their sizes are *n*_*s*1 _for *S*_*s*1 _and *n*_*s*2 _for *S*_*s*2_. Assuming that that the distributions of F_*s*1 _(*s*) and F_*s*2 _(*s*) are normally distributed with equal variance, *σ*^2^, the random variable $t_{s}=\frac{\sqrt{\frac{n_{s1}n_{s2}}{n_{s1}+n_{s2}}}\left({\bar{S}}_{s1}-{\bar{S}}_{s2}\right)}{\sqrt{\frac{\sum_{i=1}^{n_{s1}}{\left(S_{is1}-{\bar{S}}_{s1}\right)}^{2}+\sum_{j=1}^{n_{s2}}{\left(S_{js2}-{\bar{S}}_{s2}\right)}^{2}}{n_{s1}+n_{s2}-2}}}$ has a t-distribution with *n*_*s*1 _+ *n*_*s*2 _- 2 degrees of freedom.

(4) Weight each stratum-specific test statistic by the amount of data available. Dawson proposes a weight that will increase with the number of participants, *n*_*s*1 _and *n*_*s*2_, within stratum and with the number of observations per person in a given stratum, *g*_*s *_[13].

$$w_{s}=\sqrt{\frac{g_{s}n_{s1}n_{s2}}{n_{s1}+n_{s2}}}$$

For example, Table 1 demonstrates the number of subjects in each treatment arm for each stratum, where strata are defined by the number of visits each subject accumulates until dropout occurs (for this particular example, 22 subjects had 1 visit before drop-out, 17 subjects had 2 visits before drop-out, etc.). A weight for stratum 4 would be computed as $w_{s}=\sqrt{\frac{g_{s}n_{s1}n_{s2}}{n_{s1}+n_{s2}}}\Rightarrow w_{4}=\sqrt{\frac{g_{4}n_{41}n_{42}}{n_{41}+n_{42}}}=\sqrt{\frac{4*15*5}{15+5}}=3.87$. Whereas, a weight for stratum 8 is computed as $w_{8}=\sqrt{\frac{8*38*40}{38+40}}=18.97$. The weight for stratum 8 is greater than that of stratum 4 because stratum 8 consists of a greater number of subjects (78 versus 20) as well as a larger number of longitudinal time points (8 versus 4) per subject until drop-out.

**Table 1:** Example of Subject Stratification for SSS, Rows Indicate the Treatment Arms, Columns Indicate Strata and Cell Values Indicate the Number of Participant

	Strata				
Treatment	1	2	4	5	8

Placebo	10	7	15	14	38
Drug	12	10	5	14	40
Total	22	17	20	28	78

(5) Combine weighted test statistics into an aggregate statistic (Dawson, 1994).

$$\begin{array}{ccc} \left(a\right) & Z=\frac{\sum_{s=1}^{t}w_{s}t_{s}}{\sqrt{\sum_{s=1}^{t}w_{s}^{2}}}, & \text{s}=1,...,t. \end{array}$$

The aggregate statistic in equation (a) is a weighted sum of the stratum specific test statistics where the weight, *w*_*s *_is defined in Step 4 and the test statistic, *t*_*s *_is defined in Step 3. This aggregate statistic is then compared to a standard normal distribution [12].

### Modified SSS

The SSS aggregate statistic as contributed by Dawson [13] may need to be slightly modified when a t-test rather than a z-test is chosen for the stratum specific test. That is, the aggregate statistic may need to adjust for the degrees of freedom for each stratum specific t-test. One modification of SSS is to multiply each stratum specific t-test by the inverse variance of the linear combination of the t-test statistics.

Then the aggregate statistic is:

$$\begin{array}{ccc} \left(b\right) & Z=\frac{\sum_{s=1}^{t}w_{s}t_{s}}{\sqrt{\sum_{s=1}^{t}w_{s}^{2}Var\left(t_{s}\right)}}=\frac{\sum_{s=1}^{t}w_{s}t_{s}}{\sqrt{\sum_{s=1}^{t}w_{s}^{2}\frac{v_{s}}{v_{s}-2}}}, & \text{s}=1,...,t. \end{array}$$

The variable *v*_*s *_is the number of degrees of freedom associated with each stratum specific t-test statistic.

### Fisher's Combination of Probabilities from Independent Tests of Significance

The stratified summary statistic procedures described above are an example of combining independent test statistics. The statistical literature has supported many methods of combining independent data and includes combining estimates, test statistics or p-values [12,21-26]. A popular method for combining one-sided p-values was proposed by Fisher in 1950 which defines the following test statistic

$$\begin{array}{cc} \left(c\right) & \text{T=}-2\sum_{s=1}^{t}\ln\left(p_{s}\right) \end{array}$$

where *p*_*s *_is the p-value for each stratum, *s *= 1,..., *t*. The test statistic is then compared to a chi-square with 2t degrees of freedom.

The sum of *s *= 1,..., *t *independent random variables where each variable has a chi-square distribution is also a random variable that is distributed chi-square. The 'degrees of freedom' for the summed random variable is calculated by summing the degrees of freedom of each of the *s *independent random variables. Using equation c, let $T=\sum_{s=1}^{t}T_{s}$ where *T*_*S *_is distributed $X_{2}^{2}$. Given *T*_*S *_are independent: $\sum_{s=1}^{t}T_{s}∼X_{2t}^{2}$. In order to combine data using Fisher's method, p-values must be one sided. Two sided p-values may be divided by two. Without loss of generality, the Fisher's approach would always use *P*(*T *> t*) [22].

Fisher's statistic has the advantage over Dawson's SSS in that the combined p-values will follow a chi-square distribution. Combinations of test statistics will depend upon the distribution of the test-statistics themselves, for example when combining t-test statistics the test may need to be modified to account for the degrees of freedom associated with each test as demonstrated above.

### The Z Transformation Test and the Weighted Z Test

One disadvantage of the Fisher test is an asymmetrical transformation of p-values making it sensitive to data that reject the common null in contrast to data which support the null [24]. The z-transform test does not have this sensitivity [24]. The test transforms (one to one) the one-sided p-values from independent tests (*s *= 1,..., *t*) into a z-value, *z*_*s*_, from the standard normal distribution. The following statistic is then derived from the *s*, z-values

$$Z=\frac{\sum_{s=1}^{t}z_{s}}{\sqrt{t}}.$$

Under the null hypothesis, the test statistic is then compared to a standard normal distribution.

Furthermore, the Z-transformation test may be weighted according to the power of each individual test [25]. This weighted Z method has the following test statistic

$$\begin{array}{cc} \left(d\right) & Zw=\frac{\sum_{s=1}^{t}w_{s}z_{s}}{\sqrt{\sum_{s=1}^{t}w_{s}}}. \end{array}$$

If each test has equal power and is given an equal weight, then the weighted z-transform test reduces to the z-transform test. A proposed *w*_*s *_for the test includes weights that are proportional to the inverse of the error variance of each test [25]. If t-tests are used then proposed weights are the degrees of freedom for each t-test, i.e. *w*_*s *_= *v*_*s *_[25].

The standard normal deviate, *z*_*s*_, corresponds to each one tailed p-value, *p*_*s*_. Also, the *z*_*s *_will have the same sign if the effects are in the same direction but different signs if effects are in opposite directions. That is, each *z*_*s *_should have the same sign as the corresponding t-value for each test [24,25]. Once the normal deviates are computed and combined the resulting p-value of the aggregate test may be converted to either one or two sided.

## Methods

A Monte Carlo study incorporating the general design of outpatient substance abuse clinical trials was used to assess the Type I error and power of hypothesis tests of the treatment effect. Assumptions of the simulated dataset were as follows: outcome is assumed to follow a multivariate normal distribution and within unit (subject) variation was assumed to follow a compound symmetry structure. A common correlation coefficient of 0.6 was estimated from the complete cases of previous substance abuse clinical trials [27]. Outcome was assumed to follow a linear trend, with participants in both treatments groups having similar outcome at the beginning of the study and then decreasing over time. For simulations of Type I error we let *F*_*placebo *_(*y*) = *F*_*treatment *_(*y*). Data was simulated as multivariate normal with mean vector [17 16 15 14 13 12 11 10] and *σ *(*y*_*j*_) = 20 for *j *= 1,..., 8. For simulations of power we let *F*_*placebo *_(*y*) ≠ *F*_*treatment *_(*y*), the treatment effect was assumed to increase over time, i.e. the mean vector for treatment arm was set at [17 15.05 13.1 11.15 9.2 7.25 5.3 3.35] such that the power for the SSS analysis was approximately 80%. Since this is a study of longitudinal data analysis, each participant was assumed to have at least two measurements. A total sample size of n = 100 was assessed.

Missing data patterns were assumed monotonic; i.e. each subject was observed and data was recorded until withdrawal from the study and those who withdrew were not observed for the remainder of the study [28]. Missing data patterns in which subjects miss a visit and are lost thereafter are described as monotonic [27]. This complete 'loss to follow-up' gives rise to the probability that the missing data mechanism is not random and may be dependent upon observed and unobserved values of the outcome.

Several missing data mechanisms defined by their dependence on observed and unobserved values of the outcome have been classified by Rubin [9]. The specific case of monotonic missing data mechanisms for multivariate/longitudinal data has been further described by Schafer and Graham [29]. If we assume that the outcome variable, *Y*_*ij*_, can be measured for each individual, *i *= 1,..., *n *at several points in time, *j *= 1,..., *t *as defined by the design of the longitudinal study, missing data that are classified as missing completely at random (MCAR) are independent of any outcome variables and any covariates of interest. Missing at random [30] means that *Y*_*ij *_may be dependent on any of the outcomes observed until the time of the missed visit, for *j *= *m*, i.e. the missing data are dependent on outcomes *Y*_*i*1_,..., *Y*_*i*(*m*-1)_. Missing not at random (MNAR) means that *Y*_*im *_may be dependent on any outcome not observed due to missed visits. If m is defined as the time at which a subject drops out of a study and does not return, then the missing data may be dependent on any of the unobserved outcomes, *Y*_*im*_,..., *Y*_*it*_.

Missing data due to withdrawal were tested under three missing data assumptions; i.e., missing data may be considered either MCAR, MAR or MNAR with respect to outcome. In order to simulate the missing data mechanism a complete data set was simulated. The probability of drop-out was assumed to follow a logistic regression model [31-33] and was used to simulate the missing data in the complete dataset.

For example, missing data that are MAR in a longitudinal dataset are dependent on outcomes observed prior to the dropout. If we let the function *h*_*k *_(*y*_1_,..., *y*_*k*_) where *k *= 1,...,(*t *- 1) be a covariate in a logistic regression model on the probability of drop-out we will have the following logit model: $\text{logit}\left(p_{k}\right)=\log\frac{p_{k}}{1-p_{k}}=\alpha +\beta h_{k}$, where *α *is the intercept and *β *is the slope of the logit.

The function *h*_*k*_, can be defined as the latest observed measurement, i.e. *h*_*k *_(*y*_1_,..., *y*_*k*_) = *y*_*k*_. Using the latest observation in substance abuse trials may have validity since, much of the drop-out observed may be due to relapse or no change in response. Therefore, observed positive tests or high levels of cocaine (benzoylecognine) may be predictive of drop-out and the missing data mechanism can be classified as MAR. Using this function, the probability of dropout for each time point may be computed, $p_{k}=\frac{e^{\alpha +\beta h_{k}}}{1+e^{\alpha +\beta h_{k}}}$. If *β *= 0, then the missing data mechanism is MCAR.

In order to simulate a 10% missing data percentage with a MAR missing data mechanism under the null, we set *α*_13 _= -106, *α*_14 _= -105, *α*_15 _= -104, *α*_16 _= -103, *α*_17 _= -102, *α*_18 _= -101 and *β *= 2. For a 40% missing data percentage parameters were set as follows: *α*_13 _= -70, *α*_14 _= -69, *α*_15 _= -68, *α*_16 _= -67, *α*_17 _= -65, *α*_18 _= -64 and *β *= 2.

However, if the missing data mechanism is not ignorable then the logit model for each time point may be defined as: $\text{logit}\left(p_{jk}\right)=\log\frac{p_{jk}}{1-p_{jk}}=\alpha +\beta h_{k}+\gamma y_{j}$ where time is defined *j *= 1,..., *t *and time before the last observation is defined *k *= 1,..., (*t *- 1) [31,34]. If *γ *= 0 for each time point then the dropout model is MAR; whereas, if *γ *≠ 0 for each time point then the missing data mechanism is MNAR. That is, unobserved outcome may be predictive of drop-out and the missing data mechanism may be MNAR.

To simulate a 10% missing data percentage with a MNAR missing data mechanism under the null, we set *α*_13 _= -106, *α*_14 _= -105, *α*_15 _= -104, *α*_16 _= -103, *α*_17 _= -102, *α*_18 _= -101, *β *= 0 and *γ *= 2. For a 40% missing data percentage with a MNAR missing data mechanism parameters were set as follows: *α*_13 _= -70, *α*_14 _= -69, *α*_15 _= -68, *α*_16 _= -67, *α*_17 _= -65, *α*_18 _= -64 *β *= 0 and *γ *= 2. In order to simulate 40% missing data with a combination of MAR and MNAR missing data mechanisms, we set *α*_13 _= -105, *α*_14 _= -104, *α*_15 _= -103, *α*_16 _= -102, *α*_17 _= -101, *α*_18 _= -100 *β *= 2 and *γ *= 2.

Two thousand simulations were preformed for each method for missing data percentages of 10% and 40% and missing mechanisms of MCAR, MAR, a combination of both MAR and MNAR, and MNAR. To meet the standards of computation-based analysis, the optimal number of simulations was calculated using the coverage probability of 95% around the estimated Type I error probability of .05 [35]. Using this method, the simulation sample size was approximately 2,000. This simulation size also results in both Type I error estimates and power estimates which had standard errors less than or equal to .01.

Several methods of combining independent data were used to analyze each data set. Specifically, participants were stratified into mutually exclusive missingness categories. Stratum specific independent t-tests were computed using slope means for each treatment arm. Each t-statistic or p-value was weighted. Stratum specific t-statistics or p-values were then combined into an aggregate statistic and compared to the standard normal distribution. Empirical size and power for each method of analysis was compared over 2000 simulations.

### Choice of Weights

For a t-test, power should be maximized when *w*_*s *_is proportional to the noncentrality parameter of the distribution of each stratified test statistic, *Z*_*s*_, for a given model [12,16,36]. A general weight that is proportional to the non-centrality parameter is $\sqrt{\frac{n_{1}n_{2}}{n_{1}+n_{2}}}$.

A variety of weights may be chosen to increase the power of the test. Estimates of population weights may also be utilized [11]. The population weights for each stratum can be defined: $w_{s}=\frac{n_{s}}{N}$, where $\sum_{s=1}^{t}w_{s}=1$. The population weights will weight the t-tests produced from a larger proportion of the sample heavier than those with smaller sample size. Choice of weights will affect the power of the test, any weight that weights a more efficient estimate heavier than a less efficient estimate will produce a more powerful test.

Another weight may incorporate the Sum of Squares for Time. Generally t-tests are uniformly most powerful tests; however, the t-tests do not incorporate the efficiency gain by measuring participants over a number of longitudinal time points. One way to improve efficiency may be to weight each t-test by the source of variation due to time. The Sum of Squares for time may be calculated, $SSTime=kn{\sum_{j}\left({\bar{Y}}_{.j.}-{\bar{Y}}_{...}\right)}^{2}$ for each stratum and used to weight each t-test.

$$\begin{array}{cc} Z=\frac{\sum_{s=1}^{t}SSTime_{s}t_{s}}{\sqrt{\sum_{s=1}^{t}SSTime_{s}^{2}\frac{v_{s}}{v_{s}-2}}}, & \text{s}=1,...,t. \end{array}$$

## Results

Overall the results demonstrate nominal Type I error probabilities for Fisher's Method, the Weighted Z-Transform Test and Modified SSS compared to SSS (using stratum specific t-tests) under a variety of assumptions. However, SSS produced larger Type I errors compared to the other methods. Further, the modified SSS which corrects for the degrees of freedom associated with the t-tests produced tests of nominal size. Type I error probabilities showed little variation for a 10% missing rate compared to a 40% missing rate.

Table 2 demonstrates the Type I error probability under a variety of missing data percentages (10% and 40%) and mechanisms (MCAR, MAR, a combination of MAR and MNAR as well as MNAR) for all methods. Simulations for this particular table assumed a small sample size of 100, a common correlation coefficient of .6 and a simulation number of 2000. For all conditions, the Type I error probabilities of SSS are larger than those of the other methods compared. The Fisher method produces the most conservative results in terms of Type I Error; however, the differences are negligible. Finally, little variation is observed in the Type I error probabilities between the different missing data percentages and/or mechanisms.

**Table 2:** Type I Error Probabilities of Methods for Missing Data Percentages (10% and 40%) and Mechanisms

Method	Missing Percentage	MCAR	MAR	MAR/MNAR	MNAR
SSS^a^	10%	0.0920	0.0895	0.0875	0.0895
	40%	0.0945	0.1035	0.1050	0.1115
Modified SSS^b ^(Dawson Weight)	10%	0.0615	0.0580	0.0580	0.0585
	40%	0.0525	0.0520	0.0495	0.0555
Modified SSS^b ^(Population Weight)	10%	0.0600	0.0585	0.0585	0.0585
	40%	0.0535	0.0520	0.0535	0.0540
Modified SSS^b ^(SSTime Weight)	10%	0.0615	0.0590	0.0575	0.0590
	40%	0.0540	0.0535	0.0575	0.0525
Fisher^c^	10%	0.0495	0.0555	0.0495	0.0510
	40%	0.0495	0.0445	0.0505	0.0460
Weighted Z-Transform^d^	10%	0.0575	0.0595	0.0580	0.0570
	40%	0.0545	0.0550	0.0525	0.0530

*Type I error, r = .6, n = 100, simulations = 2000

*a, b, c, d indicate the methods of analysis in the text

Power for each test differed dependent on the method used as well as the missing data percentage and mechanism assumed. Table 3 demonstrates the power under a variety of missing data percentages (10% and 40%) and mechanisms (MCAR, MAR, a combination of MAR and MNAR as well as MNAR) for all methods. Simulations for this particular table assumed a small sample size of 100, a common correlation coefficient of .6 and a simulation number of 2000. Results for Table 2 demonstrate that power was generally greater for SSS compared to all other methods; however, this may be due to the inflated Type I error probabilities as previously discussed. Power was comparable across methods for the 10% missing data percentage. However, Fisher's method demonstrated a reduction in power for the 40% missing data percentage compared to modified SSS and the Weighted Z-Transform Test. Second only to SSS, the weighted Z-transform test demonstrated robustness in power for all missing data percentages and mechanisms.

**Table 3:** Power of Methods for Missing Data Percentages (10% and 40%) and Mechanisms

Method	Missing Rate	MCAR	MAR	MAR/MNAR	MNAR
SSS^a^	10%	0.8370	0.9355	0.9225	0.9355
	40%	0.5705	0.6125	0.5750	0.5600
Modified SSS^b ^(Dawson Weight)	10%	0.8165	0.9000	0.8810	0.8970
	40%	0.4445	0.3755	0.3660	0.3000
Modified SSS^b ^(Population Weight)	10%	0.8240	0.9075	0.8910	0.9075
	40%	0.4990	0.4150	0.4105	0.3850
Modified SSS^b ^(SSTime Weight)	10%	0.8220	0.8675	0.8820	0.8990
	40%	0.4770	0.3075	0.3795	0.3780
Fisher^c^	10%	0.7655	0.6940	0.6710	0.6855
	40%	0.3240	0.1505	0.1460	0.1195
Weighted Z-Transform^d^	10%	0.8265	0.9080	0.8965	0.9085
	40%	0.4990	0.4440	0.4355	0.4130

*Power, r = .6, n = 100, simulations = 2000

*a, b, c, d indicate the methods of analysis in the text

For all methods, power is decreased at least 35% for a missing data percentage of 10% versus 40%. Power is dramatically decreased for the Fisher method given a missing data percentage of 40% and a missing data mechanism of MAR or MNAR. In general, power fluctuations are observed for each missing data mechanism.

## Conclusion

The statistical literature has an abundance of methods of analysis for longitudinal datasets with missing data. This paper focuses on missing data methods which can be used for hypothesis tests of the treatment effect when the missing data pattern is monotonic. Specifically, Dawson's stratified summary statistic and several other methods of combining data were assessed and developed for analysis with missing data due to their robustness to the missing data mechanism. That is, stratifying data by the missing data pattern, computing stratum specific statistics and aggregating these statistics produces tests which have nominal Type I Error and optimal power even in the presence of nonignorable missing data [12-16]. These hypothesis tests of the treatment effect which are robust to the missing data mechanism may be applicable to the analysis of substance abuse clinical trials because missing data in substance abuse trials are predominately due to relapse and therefore the missing data may be nonignorable or dependent upon previous outcomes.

In this article, we have focused on two missing data percentages, a 10% rate and a 40% rate, with each treatment arm having similar amounts of missing data. In many clinical trials, the missing data percentage and/or mechanism may vary across treatment arm. Shih and Quan (21) demonstrate that Type I Error may be inflated when the missing data percentage differs between treatment arm and the missing data mechanism is MAR. Further simulation studies may want to focus on these variations and their effects on the Type I Error and power of hypothesis tests of the treatment effect.

This article demonstrates the impact that attrition can have on some of the statistical methods which are used for longitudinal data analysis. It should be noted that analysis should not be limited to these methods. These methods focus on testing hypotheses of the treatment effect. If the focus of a trial is on parameter estimation, a modeling approach of the missing data such as a pattern mixture or selection model may be more appropriate [11].

Furthermore, the stratified summary statistic methods possess a 'post hoc' quality. That is, we stratify on the pattern of missing data, which is not known until the data have been collected. In statistics we propose separation of the design from the analysis, i.e. the study design and analysis are specified in advance of data collection.

Although we will not know the exact pattern of missing data until all subject outcomes have been collected, it is well-known that substance abuse clinical trials are prone to high rates of attrition. Therefore, the use of missing data methods may be planned in advance of the study and may be specified in the study protocol. Furthermore, any reports of results from these analyses should be tempered with the knowledge that the analysis was dependent on the missing data pattern, which could not be fully discerned a priori.

The weighting schemes used in this paper are 'precision-based', and they weight stratum statistics with a larger amount of participants and/or more time point more than those with less. These methods seem to suggest that 'treatment works for those who work for it'. That is, we are weighting those subjects who perform better in the clinical trial more than those who perform worse (those that tend to drop-out due to relapse). However, these methods are preferred to 'complete case' analysis which drops subjects with any missing data. Also, results from this simulation study and several other studies demonstrate that these methods are robust to the missing data mechanism in terms of hypothesis testing of the treatment effect [12-16].

Further studies should investigate the robustness in Type I error and power of stratified summary statistics as well as bias and precision of the estimates of the treatment effect for these methods. Also, future studies may want to use other weighting schemes including 'bias-based' weights [37]. However, use of bias-based weights would need to be justified a priori by determining the cause and direction of the bias incurred due to attrition in substance abuse clinical trials. Given the known history of attrition in substance abuse clinical trials where much of the attrition may be contributed to relapse; bias based weighting schemes may be justifiable in this setting.

The simulations for the comparisons of missing data could also be further generalized. For these particular simulations, missing data rates were set at 10% and 40%. We chose a missing data percentage of 40% because of the known high prevalence of missing data in longitudinal substance abuse clinical trials [2-6]. However, these methods can be generalized to more intermediate missing data percentages in order to demonstrate changes in Type I error and power with a variety of missing data rates.

No matter how well-designed a clinical trial, these high attrition rates can bias the analysis of a clinical trial. Validity in the presence of missing data is often dependent upon the method of analysis selected. Specifically, inappropriate methods may produce hypothesis tests of the treatment effect without appropriate size and/or power. Therefore, it is imperative that substance abuse clinical trials prepare for inevitable missing data due to attrition. That is, this paper demonstrates the need for policy development for evidence based practice specific to the analysis of longitudinal substance abuse clinical trials in the presence of substantial drop-out. For example, given the wide variety of methods used for analysis of substance abuse clinical trials, we may want to specify that missing data methods be incorporated into the design and analysis given the unique properties of this research paradigm.

## Authors' contributions

SH conceptualized the study, carried out the Monte Carlo simulation studies and drafted the manuscript. RW participated in the statistical methods of the study, added to and edited the manuscript. RM participated in the clinical applicability of the study and edited the manuscript.
